# Overactive Bladder and Its Impact on Everyday Life: A Qualitative Study

**DOI:** 10.7759/cureus.88539

**Published:** 2025-07-22

**Authors:** Jette Sigvard Fjord, Thordis Thomsen, Susanne Vahr Lauridsen, Maria Borgstrøm

**Affiliations:** 1 Department of Urology, Copenhagen University Hospital Herlev-Gentofte, Copenhagen, DNK; 2 Department of Anesthesiology, Copenhagen University Hospital Herlev-Gentofte, Copenhagen, DNK; 3 Department of Clinical Medicine, University of Copenhagen, Copenhagen, DNK; 4 Department of Urology and Center of Perioperative Optimization, Copenhagen University Hospital Herlev-Gentofte, Copenhagen, DNK; 5 WHO-Collaborating Centre, Parker Institute, Frederiksberg-Bispebjerg Hospital, Copenhagen, DNK; 6 Department of Obstetrics and Gynecology, Copenhagen University Hospital Herlev-Gentofte, Copenhagen, DNK

**Keywords:** healthcare, impact on daily life, oab, overactive bladder, quality of life, relatives

## Abstract

Aim: The aim of this study was to explore the challenges and limitations experienced in everyday life by people living with overactive bladder (OAB).

Design*: *This is an exploratory, qualitative study with individual, semi-structured interviews

Methods: Interviews were conducted in 2021 using a semi-structured interview guide. Interviews were recorded and subsequently transcribed. Thematic analysis was used to analyse the interview text.

Results: Thirteen participants were included, eight women and five men, with an age range of 39-88 years. Three main themes emerged: (i) Organizing everyday life to accommodate the OAB, (ii) Self-taboo and shame, and (iii) Relatives' knowledge and understanding of the challenges of living with OAB. The findings indicate that OAB significantly impacts both physical and mental well-being and daily life. Participants felt limited in their daily activities and experienced poor quality of sleep. OAB prevented them from engaging in activities that they had previously enjoyed. Participants experienced a form of self-taboo and shame due to their OAB.

Conclusion*: *Participants in this study experienced OAB as an invalidating presence in multiple aspects of everyday life. Hence, they organized their daily activities to accommodate the symptoms associated with OAB. Self-taboo and shame were prevalent feelings, causing some participants to shy away from seeking professional help. Likewise, the participants’ everyday lives were affected by their relatives’ lack of understanding about OAB.

Implications for the profession and/or patient care*: *There is a need for increased focus on the consequences of OAB for those living with the condition. Interventions aiming to facilitate self-management and alleviate everyday challenges associated with the condition are warranted.

## Introduction

Overactive bladder (OAB) is a chronic disease characterized by urgency, frequency, nocturia, with or without urgency incontinence and without urinary tract infection. OAB has been shown to have debilitating effects on QoL [1-3]. People with OAB experience psychosocial burden and multiple challenges with planning their everyday life [4,5]. Few studies have explored how OAB impacts the individual [4-7], and to improve care for patients with OAB, this knowledge is important.

Background

OAB symptoms are prevalent in both men and women and are seen more frequently with increasing age [8]. Patients with OAB report lower quality of Life compared with other chronic diseases such as heart diseases, cancer, asthma and diabetes [9]. Former studies have explored patient and physician decision making in treatment of OAB [5], the impact of OAB symptoms, particularly on self-esteem, social and personal relationships [7], patients’ experiences of and perspectives on OAB symptoms, particularly their impact upon HRQL, identity and self-image, social and intimate relationships [4] and women’s perceptions of their OAB symptoms and the experience of the treatment [6]. Studies found that living with OAB causes feelings like embarrassment, distress and isolation from family and friends [5,7]. Likewise, the symptoms influence the individual’s social activities, planning of daily life, self-esteem, risk of depression and fear of urinary leakage [4]. Only one study reported the impact on everyday life and found that OAB made patients feel anxious because of constantly worrying about where to find the next toilet or the impact of OAB on intimate relationships [4]. Therefore, the aim of this study was to further explore the challenges and limitations on everyday life experienced by people living with OAB.

## Materials and methods

Design

This was an explorative qualitative study with individual semi-structured interviews. The study is reported according to the Consolidated Criteria for Reporting Qualitative Research (COREQ) [10].

Study setting and recruitment

The patients were recruited from the department of urology at Copenhagen University Hospital, Herlev/Gentofte in 2021. Patients were informed verbally and in writing about the study by a nurse in the urology outpatient department and invited to participate in the study. Those accepting participation provided written informed consent.

Inclusion criteria and sampling strategy

Patients scheduled for Botulinum toxin injections in the bladder due to lower urinary tract symptoms (LUTS), speaking Danish and mentally able to participate in interviews were included. To elucidate the research question, we used purposeful sampling and the concept of ”information power” guided the sample size [11].

Data collection

Data were collected via individual semi-structured interviews [12,13] by the first author. The interviewer had clinical experience with OAB treatment and care. However, it was ensured that there had been no prior contact between the interviewer and the participants before the planned interviews. Participants could choose to be interviewed face-to-face or by telephone. Face-to-face interviews were conducted in an office aside from the clinic which enabled a more relaxed situation. An interview guide was generated in collaboration with specialized urology nurses. The interview guide included questions about everyday life, quality of life with OAB, including social relations, limitations, quality of sleep and participants’ experiences with the healthcare system (Table 1).

**Table 1:** Example of interview questions from the interview guide: Life with an overactive bladder OAB: Overactive bladder

How do you experience your everyday life with OAB? Try to describe it to me.
Do you experience incontinence related to the overactivity? If yes, how does the incontinence effect on your everyday life?
How do you adapt your everyday life? Describe what you do?
How does the OAB influence on being with other people?
Can you talk to other people about your problem?
When was your urination when you were a child? Did you have any problems staying continent? When did it start/stop?
How long time did you have the problem before you sought help at a healthcare professional? How come it took that long time?
How did it change the way you look at/handle your disease after you decided to seek for help?
Has the referral for treatment made it easier for you to talk with other people/relatives about your situation?

The interview guide supported an interview structure while also allowing participants to unfold their experiences and thoughts. The interviewer posed probing questions on subjects brought up by participants.

Data analysis

Interviews were transcribed verbatim, and we used a reflexive thematic analysis approach as described by Braun and Clarke [14]. This method is suited to exploring participants' experiences, perceptions, and meaning-making processes and is increasingly recognised for its rigorous and flexible approach within health research.

The six analytic phases we used were: Phase 1. The first author familiarized herself with the data and read the transcripts several times, phase 2. Initial codes were identified inductively using line-by-line coding, phase 3. Themes were then constructed by grouping related codes, phase 4. Potential themes were reviewed to ensure coherence and distinction, and phase 5. We defined and named the themes to capture the essence of each. Phases 4 and 5 were done in a collaboration in the group. Phase 6. The first author drafted the manuscript and, all authors developed and discussed it collaboratively. The analytic process was recursive rather than linear, allowing themes to be refined through ongoing reflection. Critical self-awareness of the researchers’ assumptions and values were sought through regular peer-discussions to maintain reflexivity [14].

Ethical considerations

The study was approved by the Danish Data Protection Agency (j.-nr.: P-2021-67). Furthermore, the study was notified at the regional ethics committee (j.nr.: H-21002310), who declared the study exempt from formal approval according to Danish law.

## Results

Characteristics of participants

In total, 13 participants, eight women and five men, were interviewed. The age range was between 39 and 88 years. Two participants were interviewed by phone and 11 face to face. The interviews lasted between 20 and 45 min.

The thematic analysis resulted in three main themes with related sub-themes (Table 2).

**Table 2:** Main themes and related sub-themes OAB: Overactive bladder

Organizing everyday life to accommodate the OAB
Interrupted sleep affects daytime
It is a disability you must learn to live with
Self-taboo and shame
Relatives' knowledge and understanding of the challenges of living with OAB

Organizing everyday life to accommodate the OAB

Participants described how their OAB and agonizing urge to urinate dictated how they planned everyday activities. They would constantly plan one step ahead. For example, knowing the locations of toilets, wearing clothes where stains would show as little as possible, and bringing diapers and clean clothes just in case. 

”.. It’s that you think about it before you go anywhere. You know. You go to the toilet an extra time before leaving the house. Or when you are out, you unconsciously seek out where the toilets are, right? (…) It’s in the back of my mind all the time.” (Participant 6)

Participants dreamt of being able to have a social and physically active life without constant interruptions. Social interaction was challenged by interrupted conversations, having to leave the dinner table while eating, not being able to share experiences with friends and family because they constantly had to focus on their bladder, and not being able to go for longer walks without having to urinate before leaving home and several times underway. Similarly, partaking in sports was difficult, all factors resulting in the decision to opt out of activities, leaving them physically and mentally isolated.

Several participants dreamt of being able to travel again without a suitcase filled only with diapers. Travelling was difficult due to the frequent need to urinate.

”.. That I could go for longer walks, I would say, that I could have a bigger radius. That would be amazing. Also, back in time (…), before all this Corona stuff, we sometimes went to visit museums and then we found a place to eat and things like that, right? It’s just not so much fun when you must look for a toilet sign all the time, right? It takes away some of the pleasure. That’s for sure. So, I dream of being normal.” (Participant 9)

The participants expressed both psychological and physical factors that exacerbated their OAB, for example, stress, nervousness, feeling cold, hearing water running, a key in the lock and other people urinating.

”When I know - now we’re home and I’m just going to drive into the garage, then it’s like “now you are home, now you have to do something about it”. And then sometimes it’s too late and then it’s one of the times I wet myself that day. That’s how it is.” (Participant 4)

Some participants had lived with OAB since childhood. They described being bedwetters until late childhood and described how they were scolded and slapped when they wet their bed or trousers.

” (…) I think that stupid bladder has always followed me. Ah. Because I had a very hysterical mother. Ah. I was a bedwetter all the way to puberty. Ah, gradually the bigger I got - I found out that I shouldn’t wake my mother up or tell my mother, because I could just clean up myself. Otherwise, I got slapped, right? Ah. So, I think there has always been something about that bladder.” (Participant 7)

Interrupted sleep affects daytime

All participants described interrupted sleep due to frequent nightly voiding.

”I think I’ve been up… and I don’t drink after 6 PM. I drink a glass of milk or water, or sometimes maybe a beer. And ... and then I don’t drink anymore at all. Then I may eat an apple or some carrots or something later at night. Of course, there’s water in that. But still, I must get up... last night at least seven times.” (Participant 11)

Despite restricted fluid intake, participants voided several times during the night, leaving them exhausted throughout the day. For some participants, the best sleep was on the couch in the afternoon, but during the evening, it was impossible for their bladder to relax. Some were afraid they would be unable to sleep at night if they slept during the daytime.

Living with OAB

Participants described OAB as something you “get used to”. Some tried to downplay the problem by comparing it with other conditions:

 “There are people who feel worse,” or “It’s nothing you die of.”

This approach also caused some participants to refrain from seeking professional help. Those who did seek help talked about the difficulties they encountered in getting through to the health care system. For many, it was shameful to talk to their GP about their voiding problem. Likewise, for many, it was difficult to get their GP to refer them to specialized clinics.

”It’s not until now, after … I think, 10 years (…) that I’ve undergone relevant testing (…) And now I see,” but I have (OAB)”. It’s not just something I’ve been imagining (…) It’s Alpha and Omega, you know. That they (health professionals) listen to the problems you experience.” (Participant 2)

When participants finally received specialized health care, they felt acknowledged and taken seriously. Moreover, both they and their relatives understood the disease better.

For some, being diagnosed with OAB made the condition more tangible and something for which they were not personally responsible, and the diagnosis relieved their feelings of guilt and self-punishment.

Others did not perceive the diagnosis to learning to live with OAB. Rather this was up to having a positive life attitude and accepting the condition.

“I guess I’ve seen it more like a disability. Me, myself I’ve worked a lot with disabled people, right? So, I guess I’ve always done my best to tell when other people said that a person was ill. “No, it’s a disability, and they have to learn to live with it”. And I guess (…) that’s my philosophy of life.” (Participant 10)

Self-taboo and shame

Several participants compared themselves to children, old people, or outcasts who could not take care of themselves. They felt unable to control their bodies as otherwise expected of a grown-up.

”I almost have a kind of... I wouldn’t say anxiety, but I felt bad physically and I feel like a young girl, you know, who hasn’t learnt how to go to the toilet.” (Participant 2)

This participant talked about a person she had met when she was younger. A person who had wet himself and she said:

"It’s not because you think “It’s disgusting” I thought “I felt pity for this person”. And I do worry that other people look at me and think “I feel sorry for her.” (Participant 2)

Relatives’ knowledge and understanding of the challenges of living with OAB

The participants expressed that relatives could not understand how the OAB influenced them in everyday life. Even their closest relatives could still ask questions and not really understand.

”It’s so frustrating, especially in the morning where... morning or before dinner where I have to go to the toilet several times. And not even my husband who I’ve lived with for many years. He still says: “Are you sitting there again?” Then I say:” yes, I am, because you know”. And basically, nothing comes, you know. Because otherwise I would have peed in my pants, right?” (Participant 1)

Several participants described how their family and friends often knew about the voiding problem. For example, some relatives noted how frequently participants voided and therefore advised participants to seek professional help. Other participants did not want to talk to their relatives about their problem, mainly to avoid worrying them. They spared their relatives, which often made family arrangements and weekend sleepovers difficult as participants’ challenges were not spoken out loud. Some participants, on the other hand, actively involved their relatives and invited them to accompany them to consultations. To them, this was the best way to face their OAB and it helped both the patient and the relatives to understand the disease.

"My husband went with me to the consultation, and that made him realize something, right? That it wasn’t me who was just sloppy and had to pee all the time, right? That I actually had a problem.” (Participant 1)

During the Corona epidemic, some participants described immense relief due to the shutdown. Suddenly, there were no expectations about going to family gatherings or social arrangements. There was a legitimate reason to cancel.

## Discussion

Our findings show that OAB had a major impact on participants' everyday lives. The participants described how they organized their whole lives according to their OAB. They experienced poor sleep because of frequent nighttime voiding, resulting in a lack of energy during the daytime. They felt limited in enjoying social relations and activities. Self-taboo and shame associated with their OAB kept participants from reaching out to health care professionals. In addition, lack of acknowledgment in the health care system, lack of trust in treatments, and not being referred to specialized treatment accentuated their feelings of shame and self-taboo. While former qualitative studies [4,5,7] describe the negative social and psychological impact of living with OAB, our study points to the importance of being diagnosed with OAB, because this helped some participants to understand and manage the condition and further explain the impact of OAB on everyday life to relatives and friends. Having a positive attitude to life was highlighted as essential for managing and coming to terms with OAB. The extensive impact of OAB on multiple aspects of life has been reported previously [3,4, 9,15-17]. Regardless of this evidence and the prevalence of OAB, 12.8 % of women and 10.8% of men [8], our findings reinforce that shame and self-taboo continue to hinder sharing of information about symptoms and referral to relevant specialized treatment of OAB. People with OAB continue to shy away from seeking professional help, among other for fear of not being taken seriously, as also reported in other studies [4,18,19].

The lack of knowledge and shame associated with OAB may reflect the general expectation that adults are in control of their bladder and that incontinence is an inevitable part of aging [20]. Furthermore, voiding is intimate, generally an “invisible” bodily function well-hidden away [21]. Maybe this provides some explanation for OAB having an inferior place in the hierarchy of diseases. Diseases in organs in the upper body, where advanced technology, intensive or acute procedures are used, especially in younger patients, are placed high up in the hierarchy. Contrary to this, chronic diseases in the lower body, especially in elderly patients, are placed lower in the hierarchy and have less priority [22].

A limitation of the study is that participants were included from a university hospital with specialized urology treatment, which could potentially influence the transferability of the results to people with OAB consulting exclusively in the primary sector. Rigour was established by carefully following the concept of trustworthiness suggested by Graneheim and Lundman [23]. To capture experiences with challenges and limitations in everyday life from people living with OAB, both men and women were included. The variation regarding age, concomitant diseases, and ethnicity made it possible to capture a variety of descriptions and experiences, which added to the transferability of the findings to a similar setting. Due to the interviewer’s clinical experience with OAB treatment and urology nursing, the interviewer focused on allowing participants to share their experiences and remained aware of their own preconceptions when conducting the interviews and analyzing the interview data. A strength of this study was the researcher triangulation, which ensured a broader understanding of the interviews and thus contributed to dependability and credibility [10]. Another strength was the rich and nuanced data, thereby feeding into the concept of information power [11].

Implications for policy and practice

This study adds to the sparse evidence about the impact of OAB on everyday life and points to the need for further research and information campaigns to help this group of patients manage OAB to maintain high health-related quality of life. One approach could be to develop a targeted patient/patient and relative intervention combining professional and peer support and education to further understanding of the disease. Campaigns at the societal level might also enhance knowledge in the general population about OAB, symptoms, and treatment options.

## Conclusions

This study explored the challenges and limitations on everyday life experienced by people living with OAB and found that participants in this study experienced OAB as an invalidating presence in multiple aspects of daily life. Hence, they organized their daily activities to accommodate the symptoms associated with OAB. Self-taboo and shame were prevalent feelings, causing some participants to shy away from seeking professional help. Likewise, the relatives’ lack of knowledge about OAB impacted the participants' everyday lives.

## Appendices

Interview guide in English

**Table 3:** Interview guide (semi-structured) OAB: Overactive bladder

Interview guide (Semi-structured): Life with an overactive bladder
Life with OAB – everyday life and quality of life
How do you experience your everyday life with OAB? Try to describe it to me.
Do you experience incontinence related to the overactivity? If yes, how does the incontinence affect your everyday life?
How do you adapt your everyday life? Describe what you do.
How does the OAB influence being with other people?
Is there any taboo connected to incontinence? How do you experience that?
Can you talk to other people about your problem?
How do you see yourself compared to other people?
How was your urination when you were a child? Did you have any problems staying continent? When did it start/stop?
Help from health care professionals
How long did you have the problem before you did seek for help from a healthcare professional? How come it took that long?
Why did you finally choose to seek help?
How did it change the way you look at/handle your disease after you decided to seek help?
Does it feel any different/more easy/ mere difficult to talk about your disease?
How do you imagine healthcare professionals can help people in your situation in the best way? Are there anything we can do to ease the way to the healthcare system?
How can healthcare professionals help people as yourself to handle/accept your disease in an easier way?
What do you as a patient want the healthcare professionals to do for you?
Do you have a feeling that you have talked to the nurse about all the stuff that you wanted in relation to your problems?
Has the referral for treatment made it easier for you to talk with other people/relatives about your situation?
Treatment with Botox
What expectations /wishes do you have for the treatment with Botox?
Do you feel that the healthcare professionals try to understand your situation? If yes, how does it feel?
Do you have a feeling that you have talked to the nurse about everything that you wanted in relation to the treatment with Botox?

Interview guide in Danish

Livet med OAB- hverdagsliv og livskvalitet - Hvordan oplever du hverdagen/livet med overaktiv blære? Prøv at beskriv dette for mig. - Oplever du inkontinens i forbindelse med overaktiviteten? Hvis ja, hvordan påvirker inkontinens din hverdag? -  Hvordan indretter du din hverdag? Beskriv hvad du gør? - Hvordan påvirker din OAB samværet med andre mennesker? - Er der tabu der er forbundet med inkontinens? Hvordan oplever du det? - Kan du tale med andre om dit problem? - Hvordan oplever du dig selv, sammenlignet med andre mennesker? - Hvordan var din vandladning som barn? Havde du nogle problemer med at holde på vandet? Hvornår startede/stoppede dette?

Sundhedsfagliges hjælp

- Hvor længe gik du med problemet før du søgte lægehjælp? - Hvorfor gik der så lang tid? - Hvad var det der gjorde at du søgte hjælp? - Hvordan har det ændret din måde at se på/håndtere din sygdom efter du besluttede dig til af søge hjælp? - Opleves det anderledes/nemmere/sværere at snakke om din sygdom? - Hvordan kunne du forestille dig, at vi som sundhedspersonale bedst kan vi hjælpe mennesker i din situation? Er der noget vi kan gøre for at det eventuelt bliver lettere at søge hjælp? - Hvordan kan vi som sygeplejersker/læger hjælpe patienter som dig til bedre at kunne håndtere/acceptere din sygdommen? - Hvad har du som patient behov for at vi gør for dig? - Oplever du at have talt med sygeplejersken om alt det der har fyldt for dig i forbindelse med dine problemer?

- Har din henvisning til behandling gjort det nemmere for dig at tale med andre/pårørende om din situation?

Behandling med botox - Hvilke forventninger/ønsker har du til botoxbehandlingen? - Oplever du, at sundhedspersonalet prøver at forstår din situation? Hvis ja, hvordan opleves det, at sundhedspersonale forsøger at forstå din situation? - Oplever du at have talt med sygeplejersken om alt det der har fyldt for dig i forbindelse med Botoxbehandlingen?
